# Contraception Chronicles: The Gamified Approach to Postgraduate Education in Family Medicine

**DOI:** 10.12669/pjms.41.2.10239

**Published:** 2025-02

**Authors:** Syeda Ariba Hashmi, Marzia Muhammad Hanif, Ayesha Khan

**Affiliations:** 1 Syeda Ariba Hashmi, Family Medicine FCPS trainee, Department of Family Medicine, Aga Khan University Hospital, Stadium Road, Karachi, Pakistan; 2 Marzia Muhammad Hanif, Family Medicine FCPS trainee, Department of Family Medicine, Aga Khan University Hospital, Stadium Road, Karachi, Pakistan; 3 Ayesha Khan, FCPS Instructor in Family Medicine department, Department of Family Medicine, Aga Khan University Hospital, Stadium Road, Karachi, Pakistan

**Keywords:** Gamification, Contraception, Family medicine, Postgraduate training

## Abstract

Gamification involves using gaming elements in education to capture learners’ attention and motivate participation, offering innovative solutions to traditional teaching challenges. In various fields gamification enhances learning by fostering competition, collaboration and peer learning. In the realm of medical education, innovation is key to foster knowledge acquisition, critical thinking and skill development. Family physicians, dealing with comprehensive care, emphasize preventive measures like family planning and contraception. Effective contraceptive education is crucial not only for providing patient-centered reproductive healthcare but also to address the unique needs and preferences of a diverse patient population.

To make learning more engaging we incorporated the board game “Snakes and Ladders” into a didactic session for family medicine trainees. Divided into teams, they solved case-based questions linked to game progression. Feedback indicated the session was more engaging and enjoyable compared to traditional lectures and they wish to see similar teaching methods in future academic sessions.

## INTRODUCTION

Gamification refers to the application of gaming elements and techniques in the educational process, in order to capture and sustain learners’ attention and motivate them to actively participate. Gamified teaching emerges as a strategy for educational transformation, which is increasingly gaining importance. It has drawn attention in various domains such as academics, information studies, human-computer interaction and health.^1^ Gamification has offered an innovative solution to age-old challenges in teaching and learning. Many gamified learning platforms incorporate elements of competition and collaboration, nurturing a sense of camaraderie and teamwork. Such activities encourage peer-to-peer learning and knowledge sharing, as well as motivating participants to strive for excellence.

### Gamification in Medicine:

In the realm of medical education, innovation is key to foster knowledge acquisition, critical thinking and skill development. Traditional didactic methods i.e. textbooks and lectures, often struggle to fully capture and maintain learners’ attention. However, the integration of gamification offers a promising solution by leveraging the inherent appeal of games to foster development of skills and relevant competencies.

Gamified learning has become increasingly popular in medical education. Incorporating game elements in didactic sessions often involves the use of simulated scenarios that mimic real-world patient encounters. These virtual clinical scenarios allow trainees to apply theoretical knowledge in practical contexts, honing their clinical decision-making skills and helping them master the complexities of healthcare practice.

One of the key advantages of transforming learning into a game-like experience is its ability to facilitate better retention and recall of information. A variety of games have been used in medical education including “war games” to enhance high-risk clinical decision making^2^ and “escape room” for team building and timed activity.^3^ Although no study was done about the use of snakes and ladder board games in medical education, this game was used to improve knowledge about Taenia infection in school students.^4^

### Family Medicine in Pakistan:

The specialty of family medicine enjoys a special position in the medical practice of the West, serving as one of the key primary care specialties.^5^ Family physicians act as providers of first contact, catering to the medical needs of not just an individual but his entire family and the community. However, its growth has lagged behind in numerous developing countries.

Unlike many parts of the world, Family Medicine is just beginning to emerge as a specialty in Pakistan. It was formally introduced as a subject in the undergraduate curriculum as recently as 2014 by Pakistan Medical and Dental College.^6^ Postgraduate training was approved in 1992 by the College of Physicians and Surgeons, which is the premier postgraduate medical institution of Pakistan. It is a four years residency training program with a structured core curriculum designed to meet all learning needs of a family physician in training. Academic sessions, led by residents, are held once a week where all levels of trainees gather to fortify their medical knowledge, supervised by the faculty.

### Contraception in Family Medicine:

Unlike other specialities that are limited to a particular organ or disease, family physicians are the only specialists qualified to treat most ailments for people of all ages-from neonates to geriatrics. Among the myriad of services offered, family planning and contraception stands out as a vital component of preventive care. Unplanned birth can pose a significant health, social and economic challenge. By providing information about various contraceptive methods, their benefits and potential side effects, family physicians can help patients to choose the method that aligns best with their preference.

Over the years several developing countries have been suffering from high maternal and infant morbidity and mortality, Pakistan being one of them.^7^,^8^ By empowering patients to make informed decisions, we can not only prevent unintended pregnancies but can also lower the risk of maternal and infant morbidity and mortality. Therefore, contraception is an essential component of the training curriculum of post-graduate residents. This education will not only equip them with the knowledge and skills to provide comprehensive patient-centered reproductive healthcare but will also empower them to address the unique needs and preferences of a diverse patient population. Religious, cultural and socio-economic factors can significantly influence an individual’s choice and attitude towards contraception and their access to reproductive healthcare services.

Contraception is a volatile subject with numerous aspects of learning and understanding. Its contextual use while considering the medical eligibility criteria is never easy to memorize or recall. Discussing all its nitty gritty and simultaneously keeping trainees involved in a single didactic session using the conventional method of teaching is a challenge. Through repeated exposure and reinforcement of knowledge within a gaming environment, trainees can deepen and fortify their understanding of medical concepts more efficiently. As technology continues to evolve, so will the possibilities for gamification in education, enabling instructors to unlock new ways of empowering the healers of tomorrow.

### The Innovation:

We incorporated the board game “Snakes and Ladders” in our didactic session. This game has been played for centuries and its roots can be traced back to ancient India, where it was known as ‘Moksha-Patam’ and is now regarded as a worldwide classic.

### How to play:

The traditional method of playing this game is that each player rolls the dice then advances their pieces along the board’s numbered squares. They encounter snakes that pull them down and ladders that help them climb up, aiming to reach the last square 100.

### Tutorial:

How to play Snakes and Ladders.

### Description of the activity:

**Fig.1:**
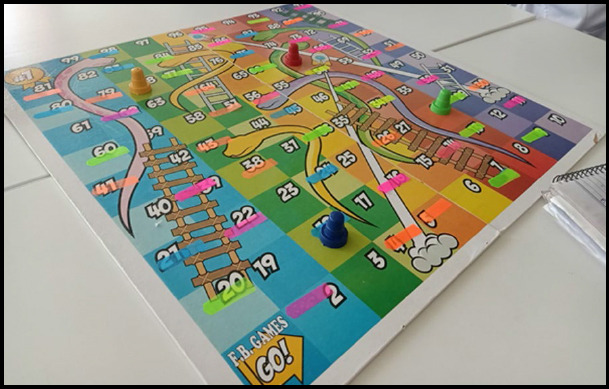
The game board.

The trainees were divided into teams. The board was color coded by the facilitator. Each team would throw the dice and move their piece. If they landed on a color coded square, then a case would open and the team needed to solve it. If they were unable to answer it or gave an incorrect answer, then the question would be passed on to the next team. (Fig.1)

### Trainees feedback:

**Fig.2:**
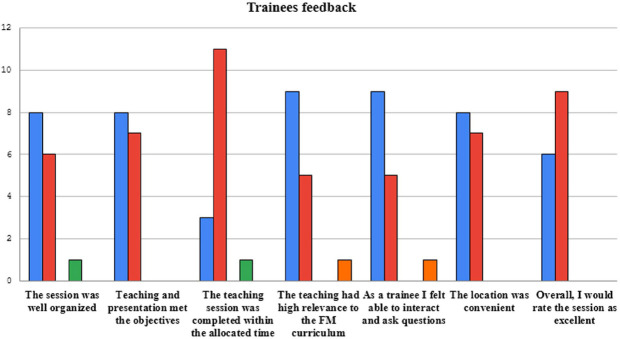
Trainees post session feedback.

This activity was well received by the residents. A post session feedback form was circulated and responses were recorded and majority of the residents thought that the session was more engaging, interactive and enjoyable as compared to the traditional didactic sessions. (Fig.2)

### Some of the comments were:

*“A very pleasant and different way of teaching, thoroughly enjoyed.”*

*“Would love to see similar teaching methods in future sessions.”*

*“A much needed breather from boring lectures.”*

### Facilitator’s feedback:

While adding games to education is both interesting and engaging, it is sometimes difficult to handle students as the fun part takes precedence over the core concept. Gamification in sessions is time consuming as it generates more discussion and engagement of the residents, therefore content of the teaching should be kept according to the allocated time. Maintaining the decorum of the session and yet making it entertaining is an art in facilitation.

### Challenges:

While gamification offers tremendous potential in education, it also presents certain challenges and considerations. Effective implementation of gamified learning experience requires creativity, careful planning, time and technical expertise. Instructors must ensure that the interactive platforms align with the learning objectives. Balancing entertainment with education is essential to make certain that the gaming environment does not sacrifice learning outcomes. Learning and teaching is a continuous process in medical education thus coming up with new and applicable ideas is not just time consuming but also requires an extra level of caution as putting all the effort and focus on gamified teaching should not compromise other clinical activities.

## CONCLUSION

As the landscape of medical education continues to transform, embracing innovative strategies like gamification holds the potential to revolutionize the way we educate and train residents. Gamification in our didactic session, as perceived by the trainees, is an encouraging strategy for impactful and effective learning. Since postgraduate medical education is demanding and competitive, therefore making this process diverting and innovative is an essential step in shaping the next generation of physicians.

### Authors Contributions:

**SAH:** Assisted in arranging the session, developed the manuscript.

**MMH:** Lead the gamified academic session, reviewed and edited the manuscript.

**AK:** Provided the idea, facilitated the session, reviewed, edited and finalized the manuscript.

All authors have read the final version and are accountable for the integrity of the study.
